# When Advisors’ True Intentions Are in Question. How Do Bank Customers Cope with Uncertainty in Financial Consultancies?

**DOI:** 10.3389/fpsyg.2017.01112

**Published:** 2017-06-30

**Authors:** Barbara Mackinger, Eva Jonas, Christina Mühlberger

**Affiliations:** Department of Psychology, Social Psychology, University of SalzburgSalzburg, Austria

**Keywords:** uncertainty, self-interest, fairness, trust, advice taking

## Abstract

When making financial decisions bank customers are confronted with two types of uncertainty: first, return on investments is uncertain and there is a risk of losing money. Second, customers cannot be certain about their financial advisor’s true intentions. This might decrease customers’ willingness to cooperate with advisors. However, the uncertainty management model and fairness heuristic theory predict that in uncertain situations customers are willing to cooperate with financial advisors when they perceive fairness. In the current study, we investigated how perceived fairness in the twofold uncertain situations increased people’s intended future cooperation with an advisor. We asked customers of financial consultancies about their experienced uncertainty regarding both the investment decision and the advisor’s intentions. Moreover, we asked them about their perceived fairness, as well as their intention to cooperate with the advisor in the future. A three-way moderation analysis showed that customers who faced high uncertainty regarding the investment decision and high uncertainty regarding the advisor’s true intentions indicated the lowest intended cooperation with the advisor but high fairness increased their cooperation. Interestingly, when people were only uncertain about the advisor’s intentions (but certain about the decision) they indicated less cooperation than when they were only uncertain about the decision (but certain about the advisor’s intentions). A mediated moderation analysis revealed that this relationship was explained by customers’ lower trust in their advisors.

## Introduction

Ninety-four-year-old Mrs X, whose cognitive and physical abilities are impaired by old age, received a sizeable amount of money from her disbursed life insurance. To reduce her uncertainty on how to reinvest the money, she consulted a financial advisor. The advisor recommended that the “optimal” solution was to put her money in a long-term investment—obviously not considering Mrs X’s age (WirtschaftsWoche, 2009). Thus, one may doubt the advisor’s trustworthiness. What was the advisor’s motive and has s/he acted in the client’s best interest or rather in her/his own interest? In 2008 the global financial crisis had swamped the Eurozone. During this time the crisis dramatically expanded in the European public sphere. It was not only centered in the attention of political leaders, public opinion, and media. Also private persons like Mrs X were involved and harmed. Especially, those who had been working with a financial advisor were now facing a dilemma: on the one hand they wanted and needed advice, but on the other hand they were not sure if they could rely on their financial advisor. In recent years the media has reported many incidents where experts did not have the knowledge about the optimal investment or at any rate did not use their knowledge to best help their customers. As a result, the questions remain—how can customers regain confidence and how can banks and financial consultants win back their customers’ trust in uncertain situations?

### Uncertainty

Uncertainty is experienced when people feel unable to predict future events or when they feel inconsistencies between important cognitions, experiences, or behaviors (Van den Bos and Lind, 2002). Uncertainty can be provoked by various situations. For example, thinking about insecure aspects of one’s self or one’s relationships to others, or losing control over one’s own life can lead to aversive and threatening feelings (Van den Bos and Miedema, 2000; Van den Bos, 2001) which people strive to reduce (Van den Bos and Lind, 2002; Heine et al., 2006; Hogg, 2007). Such uncertainty is also relevant in investment decisions where people are dependent on the financial market. Even if they choose a low risk product such as a long-term fixed capital saving, they face the risk of a decreased interest rate. Therefore, investing money is a risky decision which entails uncontrollable consequences, making one’s future unpredictable and thus, uncertain.

### Uncertainty about the Investment Decision

One way to cope with uncertainty about one’s decisions is to look for advice (for overview see Bonaccio and Dalal, 2006). Advice helps people to optimize their choice and gain confidence in their decisions (Heath and Gonzalez, 1995; Budescu et al., 2003). This is especially true for financial investment decisions, which contain high risks, in particular the risk of losing money. However, an advisor’s knowledge reduces such uncertainty and improves accuracy only if people can trust the advice they receive (Sniezek et al., 2004; Yaniv, 2004). This means that even if people decide to receive advice, they cannot be sure if the advice is of high quality.

To estimate the quality of the advice, customers use cues. One such cue is an advisor’s expertise. Harvey and Fischer (1997) showed that people are twice as likely to accept advice from experts as to accept advice from novices. In particular they found that perceived expertise predicts subsequent advice utilization, suggesting that a person’s impression of whether or not an advisor is an expert is very important to clients (de Vries and Wilke, 1995; Harvey and Fischer, 1997; Sniezek and Van Swol, 2001; Jungermann and Fischer, 2005). Thus, the perceived expertise of the advisor is a cue, which can reduce people’s uncertainty regarding their decisions. With regard to high-risk decisions, also a second cue is important – the advisor’s expressed confidence (for an overview, see Bonaccio and Dalal, 2006). If an advisor is perceived as highly confident, customers gain confidence themselves (Sniezek and Buckley, 1995; Sniezek and Van Swol, 2001; Price and Stone, 2004). As a consequence, recommendations of highly confident advisors are followed more often than those of less confident peers (Sniezek and Buckley, 1995; Sniezek and Van Swol, 2001). However, advisors often overestimate their confidence (Price and Stone, 2004; McKenzie et al., 2008) and use this overconfidence as a strategy to persuade customers (Van Swol, 2009). This phenomenon is also present in the context of financial consulting: participants confronted with a fictitious financial advisor preferred an overconfident advisor over a moderately confident advisor and even assumed the overconfident advisor to be more knowledgeable (Price and Stone, 2004). Thus, the expressed confidence of the advisor is another cue, which can reduce people’s uncertainty regarding their decisions.

Accordingly, in situations where people are uncertain regarding their decisions, customers focus on cues allowing them to gain the needed confidence for their decisions. However, in financial consultancies, people do not only have to cope with uncertainty regarding the decision but also regarding the advisor’s true intentions either to support the client or to pursue self-interest. Therefore, in addition to having to cope with *uncertainty about their investment decision* (UnD), clients also have to cope with *uncertainty regarding the advisor* (UnA).

### Uncertainty about the Advisor’s Intention

Uncertainty regarding the advisor refers to the difficulty to identify his/her true intentions. Usually, the financial advisor possesses information and knowledge that the client lacks. The lack of knowledge makes it difficult for the client to assess whether the advisor is acting in the best interest of the client. According to Principal–Agent Theory (Ross, 1973; for an overview, see Eisenhardt, 1989), agents (advisors) who pursue their own goals instead of acting in the principal’s (client’s) best interest use their scope of action to behave in a strategic way (conflicting goals). In financial consulting, the advisor might neglect to carry out all necessary actions (“hidden action”) such as searching for less risky investments, might withhold information about potential risks from the client (“hidden information”), or might hide their true intentions (“hidden intentions”) and thus, unbalanced relationships follow. The client is dependent on the advisor and thus, susceptible to deception. The less the client knows about the advisor’s actions, information, and intentions, the more the client’s uncertainty increases.

Knowing the advisor’s intentions is crucial to the client’s continued reliance on the advisor (Bonaccio and Dalal, 2009). A study by Jodlbauer and Jonas (2011) showed that customers who did not know their advisor’s true intentions but assumed their advisor to pursue self-interested intentions evaluated him/her less trustworthy. As a consequence, people were less likely to utilize the advisor’s recommendations. So how can people cope with such uncertainty?

To find answers to this question, we build on two theoretical models from justice research – the Uncertainty Management Model (UMM) and the Fairness Heuristic Theory (FHT, Van den Bos et al., 1998; Lind, 2001; Van den Bos, 2001) which might help to reduce uncertainty regarding the investment decision and uncertainty regarding the advisor.

### The Uncertainty Management Model

The Uncertainty Management Model (Van den Bos and Lind, 2002; see also Lind and Van den Bos, 2002) describes how people cope with general uncertainty. According to the UMM, uncertainty is a general and abstract concept that can also be induced in an abstract way (e.g., by thinking about uncertainty in terms of emotions or losing control or thinking about insecure aspects of one’s own life; Van den Bos and Miedema, 2000; Van den Bos, 2001). Such general uncertainty, which makes people’s future unpredictable increases people’s sensitivity to fairness cues, e.g., whether one has voice in a given situation (procedural fairness; Van den Bos and Miedema, 2000; Van den Bos, 2001). People also react with more positive affect toward fair and more negative affect toward unfair treatment (Van den Bos, 2001). Thus, applied to investment decisions, fairness may be a valuable cue when people experience general uncertainty about their decisions.

### The Fairness Heuristic Theory

The Fairness Heuristic Theory (Van den Bos et al., 1998; Lind, 2001; Van den Bos, 2001) explains how people cope with uncertainty regarding a person’s trustworthiness in an interdependent relationship, i.e., a relationship in which one is dependent on another person (e.g., employee and authority). Uncertainty regarding trustworthiness means that one does not know whether the other person will keep one’s best interests in mind (Barber, 1983). Thus, fear of exploitation and exclusion is present (Van den Bos et al., 1998; Lind, 2001; Van den Bos, 2001). In such situations, people use fairness cues to decide whether to cooperate with the other person. For example, when participants were uncertain about the trustworthiness of an authority, they showed higher commitment to a decision when they were given voice (procedural fairness) compared to no voice (Van den Bos et al., 1998). This means that fairness can compensate for people’s uncertainty and the resulting unwillingness to cooperate because of the interaction partner’s trustworthiness. However, the authors also emphasize that fairness is not the same as trust. While fairness is an evaluation whether a person acts or decides morally correct, trust is a person’s willingness to be vulnerable to the actions of another party. Therefore, trust always involves uncertainty regarding the risk of being exploited (Coleman, 1990; Moorman et al., 1992; Mayer et al., 1995). Based on this definition trust is something highly fragile (Mayer et al., 1995). The perception of trust in interdependent relationships is highly relevant when people decide whether to cooperate with a person or not (Van den Bos et al., 1998; Lind, 2001; Van den Bos, 2001). Research showed that clients who perceived self-interest on behalf of their advisors, mistrusted their advisors and were consequently less likely to accept advice (Jodlbauer and Jonas, 2011) and even showed aggressive intentions and negative attitudes toward the interaction partner (Steindl and Jonas, 2015).

Bank customers are in uncertain situations and do not know if they can trust their advisor. To investigate how people cope with such uncertainty we tested – based on the UMM and FHT – whether uncertainty increases people’s sensitivity to fairness cues and increase their willingness to cooperate with financial advisors regarding their investment decisions.

### Dimensions of Fairness

When deciding whether or not a situation is fair people seem to distinguish between different aspects of fairness (or justice^1^): the distribution of resources (distributive fairness; Adams, 1965), the provision and transparency of information (informational fairness; Bies and Moag, 1986), the procedures on which the decision is based (Thibaut and Walker, 1975; Leventhal, 1976, 1980), and whether they have been treated with respect and dignity (interpersonal fairness; Bies and Moag, 1986; for an overview, see Colquitt, 2001). These four aspects of fairness perceptions are used as cues to guide evaluation in uncertain situations (e.g., Van den Bos and Miedema, 2000; Lind, 2001; Van den Bos, 2001). Thus, the advisor’s expressed fairness may also play an important role in financial consultancies characterized by high uncertainty. Here, fairness may compensate for uncertainty.

### The Current Research

In financial consultancies people are facing those two kinds of uncertainty—uncertainty regarding the financial investment decision in general and uncertainty regarding the advisor’s true intentions. In particular in the course of the financial crisis starting in 2008, people were confronted with both uncertainties. Thus, we assume that they were facing general uncertainty about their financial decisions and were especially vigilant to financial advisors who acted in self-interested manners which increased their uncertainty about the advisors’ trustworthiness. To investigate how both uncertainties worked together during the financial crisis, we integrated the UMM and the FHT. Both theories state that people need fairness cues to regain certainty. Therefore we predicted that perceived fairness is important when any uncertainty is high and especially important when both uncertainties are high. In the current study, we investigated how the two types of uncertainty influenced people’s intended future cooperation with an advisor and whether fairness cues can promote trust and thus help to increase cooperation.

## Materials and Methods

### Procedure

Participants were invited to take part in a survey on financial consulting^2^ and asked if they had contact with a financial advisor in the last year. The data were collected in November 2009, shortly after the beginning of the financial crisis in Europe. In a questionnaire participants were asked to think about their experiences with “saving and investing money” over the last year. To answer the questions they were instructed to think about their financial service provider. If they were customers of different service providers, they were instructed to concentrate on the provider with whom they had spent the most time in the last year and they were also asked how many consultancy meetings they had.

### Participants and Procedure

Two-hundred and forty-two participants answered our questionnaire. Due to missing data on the scales cooperation intention, UnA, and trust, we had to exclude nine participants, leaving us with a final sample of 233 (16–81^3^ years old; *M*_age_ = 36.86, *SD* = 15.84; 138 women and 95 men). Our participants were either approached in a shopping mall when the University of Salzburg was having a public event there or via the experimenter’s social networks. All participants were asked to fill out a questionnaire in which they had to think about their financial service providers and indicated their agreement to several items on a scale from 1 (= not at all) to 6 (= completely).

### Measures

#### Uncertainty

We assessed two aspects of customers’ uncertainty about their financial service providers: uncertainty about their investment decisions and uncertainty about their advisor (the advisor’s strategic behavior)^4^.

#### Uncertainty about Investment Decisions (UnD)

This measure focused on the uncertainty about investment decisions (whether they had made the right decision, e.g., regarding the choice of service provider or product). Five items asked about the success of the participants’ investment (e.g., “Thinking about my investment in the last year raises the feeling that I reached the “right” decision regarding my financial service provider” inverted; α = 0.81, see complete questionnaire in the appendix).

#### Uncertainty about the Advisor (UnA)

Participants were asked to think about the behavior of their financial advisor and describe his/her behavior with the help of the following items: the strategic behavior scale covered the three agency problems described above [hidden intention: e.g., “In situations where our interests were in conflict the advisor focused on his/her interests,” seven items; hidden information: e.g., “I got the impression that my advisor did not communicate essential information about the protection of the money (e.g., a capital-back guarantee),” five items; hidden action: e.g., “My advisor handed over written information about my investment product after the contract conclusion (e.g., information with risk details),” seven items]. For further analyses we combined the subscales to produce a general uncertainty scale about the advisor (α = 0.93; see complete questionnaire in the appendix).

#### Fairness

Participants evaluated their advisor’s fairness behavior with items from the German version (Maier et al., 2007) of the Organizational Fairness Scale (Colquitt, 2001). The items were adapted to the financial advisor situation. Two items were additionally developed and one original item^5^ was excluded. Procedural fairness was assessed with eight items (e.g., “Have you been able to express your views and feelings during consultations with your advisor?”). Distributive fairness was represented with three items (e.g., “How appropriate are your returns considering the amount of money you invested?”). Informational fairness was measured with seven items (e.g., “Has your advisor explained the procedures thoroughly?”) and interpersonal fairness with four items (e.g., “Have you been treated with dignity by your advisor?”). In the following calculations we combined these subscales to a general fairness scale (α = 0.94; see complete questionnaire in the appendix).

#### Trust

Trust in the financial advisor was measured with items from a questionnaire designed by Schoorman and Ballinger (2006) with the main focus on the willingness to yield control to the advisor and the customers’ accepted vulnerability. Five items were translated and adapted to the financial service sector (e.g., “I would be willing to let my advisor have complete control over my future investment decisions”; α = 0.56^6^; see complete questionnaire in the appendix).

#### Dependent Variable—Cooperation Intention

This scale measured the intention of the participants to further use the consulting services of the current financial advisor (e.g., “I will still use the services of my financial advisor in the future”; five items, α = 0.82; see complete questionnaire in the appendix). For the means, standard deviations, and intercorrelations of all measured variables, see **Table 1**.^7^

**Table 1:** Intercorrelations between all measured variables.

	*M*	*SD*	1	2	3	4	5
(1) Uncertainty about the Decision (UnD)	2.80	1.08	1				
(2) Uncertainty about the Advisor (UnA)	2.51	0.94	0.573^∗∗^	1			
(3) Fairness	4.46	0.84	-0.640^∗∗^	-0.771^∗∗^	1		
(4) Trust	3.79	0.91	-0.449^∗∗^	-0.680^∗∗^	0.620^∗∗^	1	
(5) Cooperation Intention	4.61	1.10	-0.565^∗∗^	-0.769^∗∗^	0.718^∗∗^	0.646^∗∗^	1

*Ratings were made on a six-point scale. ^∗∗^*p* < 0.01, two-tailed.*

## Results

According to the two theoretical models dealing with uncertainty—UMM (Lind and Van den Bos, 2002; Van den Bos and Lind, 2002) and FHT (Van den Bos et al., 1998; Lind, 2001; Van den Bos, 2001)—fairness plays a particularly important role when uncertainty is high. Therefore, we tested how the different types of uncertainty in combination with fairness influenced people’s decision regarding future cooperation with the advisor.

### Multiple Regression Analysis

We conducted a multiple regression including three continuous predictors (UnA, UnD, and fairness as moderator; all standardized), and all of their interactions regarding the dependent variable intention to cooperate (Hayes, 2013; Model 3). We used a 95% bias corrected bootstrap confidence interval (95% BCCI) and 5,000 bootstrap samples. High values refer to one standard deviation above and low values to one standard deviation below the standardized values for the respective variable.

The analysis revealed significant main effects of UnD (β = -0.17, *SE* = 0.06, *t*(225) = -2.65, *p* = 0.009, [95% CI: -0.29 to -0.04]), UnA (β = -0.58, *SE* = 0.07, *t*(225) = -8.37, *p* < 0.001, [95% CI: -0.72 to -0.45]), and perceived fairness (β = 0.25, *SE* = 0.08, *t*(225) = 3.36, *p* = 0.001, [95% CI: 0.11 to 0.40]) indicating that all three variables (high UnD, high UnA, and low fairness) decreased people’s cooperation intentions.

**FIGURE 1:**
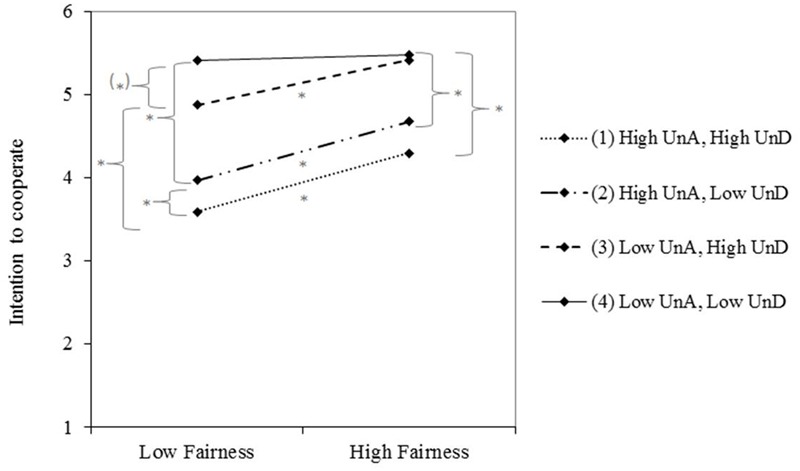
The effects of uncertainty about the advisor (UnA), uncertainty regarding the investment decision (UnD), and fairness on people’s intention to cooperate with the advisor. Plotted values reflect intention to cooperate at one standard deviation below and above the standardized values for fairness. Significant slopes are marked with asterisks. Higher values reflect higher intention to cooperate.

In addition and most importantly, the three-way interaction between UnA, UnD, and fairness was significant, β = -0.06, *SE* = 0.03, *t*(225) = -2.19, *p* = 0.030, [95% CI: -0.11 to -0.01]. Simple slopes indicated that when UnA and UnD were low, high and low fairness did not make a difference regarding people’s intentions to cooperate (*p* = 0.773). However, when any kind of uncertainty was involved (high UnA or/and high UnD), high fairness led to more cooperation intentions than low fairness (all *p*s ≤ 0.041). Furthermore, when both uncertainties were high, people had the lowest intentions to cooperate in both the low and high fairness conditions (see **Figure 1**).

Moreover, the regression weights show that the weight for UnA was higher than for UnD. This suggests that UnA had a larger impact on client’s cooperation intentions than UnD. In all conditions, whether fairness and/or UnD were high or low, high UnA always led to lower cooperation intentions than low UnA (all *p*s < 0.001). In contrast, high UnD (compared to low UnD) led to lower cooperation when UnA was high and fairness low (*p* = 0.023). See **Table 2** for a summary.

**Table 2:** Effects of the moderated regression analysis (Hayes, 2013, Model 3).

	Coefficient β	*SE*	*t*	*p*	LLCI	ULCI
UnD	-0.17	0.06	-2.65	0.009	-0.29	-0.04
UnA	-0.58	0.07	-8.37	<0.001	-0.72	-0.45
Fairness	0.25	0.08	3.36	0.001	0.11	0.40
UnD × Fairness	0.06	0.07	0.91	0.364	-0.07	0.19
UnA × Fairness	0.10	0.06	1.64	0.102	-0.02	0.21
UnD × UnA	-0.02	0.07	-0.24	0.812	-0.15	0.12
UnD × UnA × Fairness	-0.06	0.03	-2.19	0.030	-0.11	-0.01

Additionally, we performed separate moderation analyses for the four fairness dimensions and found significant three-way interactions for the procedural fairness dimension, and marginal significant three-way interactions for the distributive and interpersonal fairness dimensions, all pointing into the same direction (procedural fairness × UnA × UnD, β = -0.06, *SE* = 0.03, *t*(225) = -2.19, *p* = 0.030; distributive fairness × UnA × UnD, β = -0.05, *SE* = 0.03, *t*(225) = -1.78, *p* = 0.076; interpersonal fairness × UnA × UnD, β = -0.06, *SE* = 0.03, *t*(225) = -1.67, *p* = 0.096; see **Tables 3–6**). This suggests that especially procedural fairness is important to compensate for the uncertainties.

**Table 3:** Effects of the moderated regression analysis including procedural fairness (Hayes, 2013, Model 3).

	Coefficient β	*SE*	*t*	*p*	LLCI	ULCI
UnD	-0.20	0.06	-3.25	0.001	-0.32	-0.08
UnA	-0.67	0.06	-10.61	<0.001	-0.79	-0.55
Procedural Fairness	0.15	0.06	2.30	0.021	0.02	0.27
UnD × procedural Fairness	0.08	0.07	1.28	0.201	-0.05	0.21
UnA × procedural Fairness	0.08	0.06	1.34	0.183	-0.04	0.20
UnD × UnA	-0.02	0.06	-0.32	0.754	-0.15	0.11
UnD × UnA × procedural Fairness	-0.06	0.03	-2.19	0.030	-0.12	-0.01

**Table 4:** Effects of the moderated regression analysis including distributive fairness (Hayes, 2013, Model 3).

	Coefficient β	*SE*	*t*	*p*	LLCI	ULCI
UnD	-0.15	0.06	-2.44	0.015	-0.28	-0.03
UnA	-0.69	0.06	-11.89	<0.001	-0.80	-0.58
Distributive Fairness	0.17	0.06	2.80	0.006	0.05	0.29
UnD × distributive Fairness	0.08	0.06	1.41	0.159	-0.03	0.19
UnA × distributive Fairness	0.12	0.06	2.00	0.047	0.00	0.24
UnD × UnA	0.01	0.06	0.12	0.902	-0.11	0.12
UnD × UnA × distributive Fairness	-0.05	0.03	-1.78	0.076	-0.11	0.01

**Table 5:** Effects of the moderated regression analysis including interpersonal fairness (Hayes, 2013, Model 3).

	Coefficient β	*SE*	*t*	*p*	LLCI	ULCI
UnD	-0.21	0.06	-3.43	<0.001	-0.33	-0.09
UnA	-0.61	0.06	-9.63	<0.001	-0.74	-0.49
Interpersonal Fairness	0.22	0.07	3.21	0.002	0.08	0.35
UnD × interpersonal Fairness	0.01	0.07	0.13	0.896	-0.13	0.15
UnA × interpersonal Fairness	0.01	0.06	0.22	0.829	-0.11	0.13
UnD × UnA	-0.11	0.05	-2.16	0.032	-0.20	-0.01
UnD × UnA × interpersonal Fairness	-0.06	0.03	-1.67	0.096	-0.12	0.01

**Table 6:** Effects of the moderated regression analysis including informational fairness (Hayes, 2013, Model 3).

	Coefficient β	*SE*	*t*	*p*	LLCI	ULCI
UnD	-0.15	0.06	-2.43	0.016	-0.27	-0.03
UnA	-0.58	0.07	-7.94	<0.001	-0.72	-0.43
Informational Fairness	0.25	0.08	3.24	0.001	0.10	0.40
UnD × informational Fairness	-0.01	0.06	-0.18	0.859	-0.14	0.11
UnA × informational Fairness	0.10	0.06	1.68	0.094	-0.02	0.22
UnD × UnA	-0.05	0.07	-0.79	0.428	-0.18	0.08
UnD × UnA × informational Fairness	-0.04	0.03	-1.43	0.154	-0.10	0.02

To test whether a loss of trust in the advisor was responsible for the stronger influence of UnA compared to UnD on cooperation intentions we explored the role of trust as a mediating variable between the two types of uncertainty and cooperation intentions.

### Multiple Regression Analysis

We conducted a mediated moderation analysis using PROCESS SPSS macro (Hayes, 2013; Model 11) with the three standardized predictors UnA (as independent variable), UnD, and fairness (as moderator), trust (as mediator), and the dependent variable intention to cooperate. We used a 95% bias corrected bootstrap confidence interval (95% BCCI) and 5,000 bootstrap samples. The three-way interaction between UnA, UnD, and fairness on trust was marginally significant, β = 0.06, *SE* = 0.03, *t*(225) = 1.97, *p* = 0.050, [95% CI: 0.00 to 0.12]. Trust had a significant effect on cooperation intentions, β = 0.25, *SE* = 0.06, *t*(230) = 4.12, *p* < 0.001, [95% CI: 0.13 to 0.37]. The direct effect of UnA on cooperation intentions was significant as well, β = -0.67, *SE* = 0.06, *t*(225) = -11.09, *p* < 0.001, [95% CI: -0.79 to -0.55].

For the slopes, we found that trust mediated the conditional indirect effect of UnA on cooperation when UnD was high and fairness was low, β = -0.13, *SE* = 0.05, [95% CI: -0.25 to -0.06], as well when UnD was high and fairness was high, β = -0.16, *SE* = 0.06, [95% CI: -0.30 to -0.07], and when UnD was low and fairness was high β = -0.13, *SE* = 0.04, [95% CI: -0.23 to -0.07]. Thus, the analyses revealed a negative association with the customers’ intention to cooperate in future in three conditions. However we did not observe that trust mediated the indirect effect of UnA on cooperation when UnD and fairness were both low, β = -0.05, *SE* = 0.04, [95% CI: -0.15 to 0.03]. Please see **Table 7** for all slopes. This moderated mediation analysis shows that the negative relation between UnA and intention to cooperate can be explained via the mediator trust, which means that UnA leads to loss of trust which can better explain the reduced cooperation intention. Only when customers are confident in their decision (low UnD) and perceive low fairness, trust in the advisor does no longer seem to be involved in the process between UnA and intention to cooperate.

**Table 7:** Trust mediating the conditional indirect effects of X (UnA, Fairness, UnD) on Y (Cooperation Intention) at values of the variables (Hayes, 2013, Model 11).

UnA	UnD	Fairness	Coefficient β	*SE*	LLCI	ULCI
	-1 SD	1 SD	-0.05	0.04	-0.15	0.03
	-1 SD	+1 SD	-0.13	0.04	-0.23	-0.07
	+1 SD	-1 SD	-0.13	0.05	-0.26	-0.06
	+1 SD	+1 SD	-0.16	0.05	-0.30	-0.08

**Fairness**	**UnD**	**UnA**	**Coefficient β**	***SE***	**LLCI**	**ULCI**

	-1 SD	-1 SD	0.20	0.05	0.11	0.32
	-1 SD	+1 SD	0.08	0.06	-0.04	0.21
	+1 SD	-1 SD	0.06	0.07	-0.06	0.20
	+1 SD	+1 SD	0.03	0.04	-0.04	0.12

**UnD**	**UnA**	**Fairness**	**Coefficient β**	***SE***	**LLCI**	**ULCI**

	-1 SD	-1 SD	0.20	0.11	-0.02	0.42
	-1 SD	+1 SD	-0.02	0.06	-0.14	0.09
	+1 SD	-1 SD	0.01	0.05	-0.08	0.11
	+1 SD	+1 SD	-0.08	0.10	-0.24	0.15

## Discussion

We opened this paper by referring to the dilemma faced by financial consulting customers. On the one hand, customers want and need advice to reduce uncertainty regarding their risky decisions. On the other hand, they have to deal with uncertainty regarding the advisor’s true intentions, i.e., whether they pursue their own interests instead of the clients’ best interests. This dilemma describes two different forms of uncertainty: uncertainty regarding investment decisions and uncertainty regarding the advisor’s intentions. The current study examined customers’ intended future cooperation with an advisor when they were facing these two forms of uncertainty. Furthermore, as research has shown that in uncertain situations people need cues to regain their confidence, we introduced fairness as an important predictor for cooperation in consultancies.

We based our study on the UMM and the FHT predicting that people react more strongly to fair treatment when uncertainty salience is high. Therefore, we suggested that perceived fairness is important when any uncertainty is high and especially important when both uncertainties are high. In the present study we asked participants to think about their experiences with “saving and investing money,” assessed their uncertainty regarding the decision and regarding the advisor’s intention, and their perception of fairness in the consultancy. Our findings indicated that whenever people were uncertain in a consultancy, whether uncertain about the decision or/and advisor, their intentions to cooperate with the advisor increased with perceived fairness. Moreover, both uncertainties together led to the lowest intentions to cooperate.

Interestingly, a higher regression weight for UnA than for UnD indicated that in our study, UnA had a larger impact on client’s cooperation intentions than UnD. Thus, high UnA seems to be most costly for financial advisors regarding the customers’ intention to cooperate in the future. High experienced UnA seems to be especially detrimental for the relationship between the financial advisor and his/her customer. What changes in the relationship between customers and their advisors when they are confronted with uncertainty regarding the advisor’s intention, i.e., when people face the risk of being exploited by their advisor? The mediation analysis suggests that trust can help to explain this process: because clients mistrust that their advisors act in the client’s best interest and instead pursue their own goals, they refrain from cooperating with the advisor. However, if people want to work together in interdependent relationships, they need to trust each other (Van den Bos et al., 1998; Lind, 2001; Van den Bos, 2001). If customers, like Mrs X, face the dilemma that they need advice, but at same time are uncertain about their advisor’s intention, they have to ask themselves if they can trust the advisor. When people are uncertain about another person’s trustworthiness, trusting this person makes them even more vulnerable and thus, even more uncertain (Deutsch, 1962; Coleman, 1990; Moorman et al., 1992; Mayer et al., 1995).

In the current article, we have introduced fairness as a crucial cue in uncertain consultancies. Fairness is a cue displayed in the social interaction itself and thus differs from variables such as the advisor’s expertise or confidence which stem from the classic Judge-Advisor Research (for overview Bonaccio and Dalal, 2006). According to the input-process-output model proposed by Bonaccio and Dalal (2006), expertise and expressed confidence of an advisor are both individual level inputs. Fairness is a process variable happening in the interaction between people. It consists of different dimensions which should be taken into account in consultancies because customers pay attention to more than just fair returns on the investment of their money (distributional fairness). They also wish to be treated with dignity and respect (interpersonal fairness), to receive sufficient information (informational fairness), and to understand how decisions are made (procedural fairness; for an overview, see Colquitt, 2001). According to our results, people need fairness cues if they perceive high uncertainty. In our study procedural fairness seemed to be especially important. Thus, customers who had the feeling that they could participate in the investment decision process increased their intention to cooperate with the advisor.

### Theoretical Implications

Prior research has established FHT and UMM to explain how persons cope with these two types of uncertainty. Studies have supported each of these theoretical frameworks and the two kinds of uncertainty have been investigated separately. Thus, previous studies found fairness cues as especially helpful in highly general uncertain situations (UMM) or authority-oriented based situations (FHT). The current results expanded this line of reasoning by taking into account both uncertainties together and found that both are important in explaining cooperation intentions but that uncertainty regarding the advisor was more influential. However, they did not reinforce each other. This might be different in other consultancy situations. For example in health-related contexts – when people ask themselves what the best decision is and whether they can trust their advisors but they are anxiously aroused because they worry about their health – cues that induce mistrust might lead to overreactions. A potential mediator in this situation might be perceived loss of control. In future research, it would be fruitful to investigate the influence of different kinds of uncertainties and their relationship in various advisor–client interactions and to shed light on relevant mediators.

### Practical Implications

Resulting from the economic crisis, people seem to be highly aware of both types of uncertainty (UnA and UnD) and even more vigilant to advisor’s trustworthiness. Therefore, since then, bank customers may evaluate the financial consultancy through the eyes of uncertainty. Consequently, it is important for banks to develop strategies that help their customers overcome this uncertainty. Our findings suggest that fair treatment might be such a strategy. Fairness increased people’s intentions to cooperate even when uncertainty was high. Nevertheless, it is essential to not understand fairness as a substitute which can easily eliminate people’s uncertainty. Rather, our findings indicated the lowest willingness to cooperate with the advisor when UnA and UnD was high. Fairness slightly improved the willingness but did not set people’s willingness back to baseline (when there was no uncertainty present). Thus, the way how advisors treat their customers and explain their products is critical and should not be neglected in the day-to-day running of a financial business. Fair treatment during consultancy is one way to help customers to reduce uncertainty and regain trust in their financial advisors.

### Limitations and Future Directions

An advantage and disadvantage at the same time is that we collected our data in the field. On the one hand, a limitation of our research is that we could not control for further variables (loss of money, risk taking, etc.), on the other hand and at the same time it may be an advantage because we were able to directly examine how people cope with the insecure situation after the economic crisis. Moreover, manipulating a general uncertainty (UMM) and personal uncertainty (FHT) independently of each other in the laboratory could have helped to understand the interplay between different types of uncertainty and the role of fairness in this interplay. It would be important for future research to investigate different types of uncertainty in combination with the risk of losing real money (e.g., provide investment products with high vs. low risk) in the controlled environment of the laboratory. For future research it would also be interesting to investigate the long-term process of uncertainty and the reconstruction of trust in the advisor. Uncertainty regarding the advisor’s intentions might lead to mistrust, and fair treatment might be a way to regain trust. We are aware that we only measured people’s intention to cooperate in the future with the advisor, which is not the same as real behavior. Nevertheless, we believe that future studies might benefit from our findings and we hope that researchers further investigate the positive effect of fairness on cooperation in uncertain financial conditions.

## Conclusion

Uncertainties are part of our daily lives and especially part of our social interactions. In particular in financial consultancies, where uncertainty regarding an investment decision and uncertainty regarding the advisor’s intentions are high, we need cues helping us to deal with those uncertainties. We identified fairness as one cue helping people to compensate for uncertainty. Furthermore, our results indicate that customers’ uncertainty about the advisor is most costly for the customers’ intention to cooperate in future. However, our further analysis could identify loss of trust as a mediator, which can explain why customers facing high uncertainty about the advisor are not willing anymore to cooperate with their financial advisor.

We are grateful to Barbara Grall for helping in the data collection and to Thomas Scherndl for his statistical advice.

## Ethics Statement

Although this study used human subjects, the approval of an ethical committee does not seemed necessary. The study was only about non-intrusive issues.

## Author Contributions

BM and EJ developed together the research question, hypothesis and the research design. BM and CM did mainly the data analysis; together we did the writing of the manuscript.

## Conflict of Interest Statement

The authors declare that the research was conducted in the absence of any commercial or financial relationships that could be construed as a potential conflict of interest.

## Footnotes

1 In the literature, primarily known as distributive, informational, interpersonal, and procedural justice (e.g., Colquitt, 2001).

2 Our survey took place in November 2009; therefore uncertainty regarding investment should have been especially salient to participants.

3 Because of the financial practices in Austria, we also included 16- and 17-year old participants in the analyses. In Austria it is absolutely common for kids to have their own saving account (typically at the same financial institution as their parents) and when they turn to become teenagers, for example at the age of 16, they get a phone call once a year where they get invited to a financial consultancy. Teenagers under 18 years are already an important group of customers and get in contact with financial institutes regularly (in legal terms teenagers aged 14 and older become already contractually capable to the maximum content of their income). Additionally one of the most popular forms of saving is a so called “Bausparer” which is a long-term saving (6 years) where the government also pays a bonus. A lot of young people use this to afford, e.g., a driver’s license, motorbike or car.

4 In a confirmatory factor analysis including the two aspects of uncertainty (UnD: 5 items; UnA: 19 items), we compared a one-factor to a two-factor model. This analysis yielded a significant difference [one-factor: χ^2^(252) = 723.78, two-factor model: χ^2^(251) = 620.71), *p* < 0.001], indicating that the two-factor model is more tenable than the one-factor model.

5 “To what extent does this result reflect the effort you carried out for the company?” This original item was excluded because the transfer in the consultancy context did not work. We added two items extending the subscale informational justice. They are marked in the questionnaire in the appendix as IFG2a and IFG3a.

6 We used the trust scale introduced by Schoorman and Ballinger (2006). As two items from the original questionnaire could not be adapted to the context of the financial consultancy (“I feel comfortable being creative because my advisor understands that sometimes creative solutions do not work”; “Increasing my vulnerability to criticism by my supervisor would be a mistake”), we deleted those two items. Unfortunately, Cronbach’s alpha turned out to be very low (α = 0.55). However, this is according to some authors (e.g., Peterson, 1994) still in the acceptable range.

7 We also measured their emotions with the help of the PANAS (Watson and Clark, 1988) to which we added some additional items (e.g., uncertain, skeptical etc.) and individual differences in emotional uncertainty with the Uncertainty Response Scale (Greco and Roger, 2001). Individual emotional uncertainty did not relate significantly to the intention to cooperate, *r*(234) = -0.020; *p* = 0.761.
