# Supplementary material for: When Advisors’ True Intentions Are in Question. How Do Bank Customers Cope with Uncertainty in Financial Consultancies?
Source: Front Psychol. 2017 Jun 30;8:1112. doi: 10.3389/fpsyg.2017.01112 (PMC5492801; doi:10.3389/fpsyg.2017.01112)
Supplement: Supplementary file 1 [file Data_Sheet_1.pdf]

Appendix

4.1. Fairness Questionnaire

	**Procedural Justice**
PG 1	To what extent have you been able to express your views and feelings during the consultancy procedure?
PG 2	To what extent have you had influence over the agreed terms within the consultancy procedure (e.g. interest rates, duration etc.)?
PG 3	To what extent have you affected the achieved profit?
PG 4	To what extent has the procedure of the consultancy been applied consistently?
PG 5	To what extent has the procedure of the advisor been free of bias?
PG 6	To what extent has the procedure of the consultancy been based on accurate information?
PG 7	To what extent have you been able to disagree with provided recommendations?
PG 8	To what extent has the consultancy procedure upheld ethical and moral standards?

	**Informational Justice**
IFG 1	To what extent has the advisor been honest in his/her communication with you?
IFG 2	To what extent has your advisor provided accurate information about the procedure?
IFG 2a	To what extent has your advisor provided accurate information about the product?
IFG 3	To what extent have the explained procedures been thorough?
IFG 3a	To what extent have the explained products been thorough?
IFG 4	To what extent has your advisor communicated details in a timely manner?
IFG 5	To what extent has the advisor seemed to tailor his/her communications to your specific needs?

	**Interpersonal Justice**
IPG 1	To what extent have you been treated in a polite manner?
IPG 2	To what extent have you been treated with dignity by your advisor?
IPG 3	To what extent have you been treated with respect by you advisor?
IPG 4 umpol.	To what extent has your advisor refrained from improper remarks or comments?

	**Distributive Justice**
DG1	To what extent does the outcome reflect the effort you have put into the consulting procedure?
DG 2	To what extent is your profit appropriate for the money you invested?
DG3	To what extent is the outcome of your investment in proportion to the risk?

4.2. Intention to cooperate Questionnaire

	**Intention to cooperate in future**
Ko_1 recode.	I think about canceling existing investment products (saving banks book, Bausparer, life assurance, etc.).
Ko_2	I am going to rely on future recommendations of my advisor.
Ko_3	I am going to make use of the consultancy service of my advisor in future.
Ko_4 recode	I am going to change my financial advisor in the near future.
Ko_5	I am going to place my money again with this bank (where my advisor is working).

4.3. Uncertainty about the Advisor Questionnaire

	**Hidden Action**

H_Act_1<br><br>recode	My advisor does everything in his/her power to support me best in my decision process.
H_Act_2<br><br>recode	In the beginning the advisor inquired my situation (e.g. present financial situation, current expenses and receipts, saving activity).
H_Act_3<br><br>recode	My advisor asked for my wishes and goals (e.g. short-, medium-, long-term life planning and capital requirement, risk appetite, need for advice) before he/she made recommendations.
H_Act_4<br><br>recode	I have the impression that the investment recommendation of my advisor is ideal for my needs (period, risk etc.).
H_Act_5	I got written documents only after the conclusion of contract (e.g. information with notes on risk).
H_Act_6	I have the impression that certain actions/recommendations are rather in the advisor’s interest.
H_Act_7	I have the impression that my advisor recommended products which he/she is familiar with – even when they do not meet my needs ideally.

	**Hidden Intention**
H_Int_1<br><br>recode	I have the impression that my advisor gave recommendations only in my best interest.
H_Int_2	In situations where my advisors and my interests were in conflict, he/she aligned with his/her own interests.
H_Int_3	I have the impression that my advisor wanted to push me to a conclusion of contract.
H_Int_4	I have the impression that my advisor hid information on purpose.
H_Int_5	I have the impression that my advisor behaved sincerely (e.g. risk disclosure).

recode	
H_Int_6	I have the impression that my advisor made recommendations which are primarily in his/her best interest.
H_Int_7 recode	I have the impression that my advisor uses his/her knowledge to support me in an ideal way.

	**Hidden Information**
H_Inf_1 recode	My advisor informed me comprehensively about different investment products (saving banks book, life assurance, Bausparer, funds, stocks, etc.).
H_Inf_2 recode	I have the impression that my advisor gave me all important information about arising costs and fees.
H_Inf_3 recode	I have the impression that my advisor gave me all important information about the different risks which go along with different decisions (change of interest rates and courses, currency fluctuations).
H_Inf_4	I have the impression that my advisor withheld essential information about commitment period, termination options or sales opportunities (and anticipated losses).
H_Inf_5	I have the impression that my advisor did not communicate information about the protection of customers' monies (capital guarantee).

4.4. Uncertainty about the investment decision Questionnaire

	**Uncertainty about the investment**
	**Every time I am thinking of my savings/ financial investments….**
U_1 recode	….I have the impression my money is good invested.
U_2	….I am feeling nervous and uncertain.

U_3 recode	…I have the impression that I invested my money with the “right” investment service provider.
U_4 recode	…I am convinced that I found the right mix of risk and safety.
U_5	…I am unsecure, if the profit will arrive with this form of investment.

4.5. Trust Questionnaire (Schoorman and Ballinger, 2006)

	**Uncertainty about the investment**
T_1	My advisor keeps my interests in mind when making decisions.
T_2	I would be willing to let my advisor have complete control over my future in this company.
T_3	If my advisor asked why a problem occurred, I would speak freely even if I were partly to blame.
T_4 recode	It is important for me to have a good way to keep an eye on my supervisor.
T_5 recode	If I had my way, I wouldn’t let my advisor have any influence over decisions that are important to me.
